# “Not just a normal mum”: a qualitative investigation of a support service for women who are pregnant subsequent to perinatal loss

**DOI:** 10.1186/s12884-016-1200-9

**Published:** 2017-01-05

**Authors:** Pamela Meredith, Trish Wilson, Grace Branjerdporn, Jenny Strong, Laura Desha

**Affiliations:** 1 Occupational Therapy, School of Health and Rehabilitation Sciences, The University of Queensland, St Lucia, QLD 4072 Australia; 2 Bereavement Support Service, Mater Mothers Hospital, South Brisbane, QLD Australia

**Keywords:** Bereavement, Birth, Midwifery, Perinatal loss, Qualitative research, Subsequent pregnancy, Service evaluation

## Abstract

**Background:**

Following previous perinatal loss, women in a subsequent pregnancy may experience heightened emotions, such as anxiety and fear, with a range of longer-term implications. To support these women, the Mater Mothers’ Bereavement Support Service in Brisbane, Australia, developed a Pregnancy After Loss Clinic (PALC) as a specialised hospital-based service. The present study investigated the experiences of mothers with previous perinatal loss in relation to: (a) their subsequent pregnancy-to-birth journey, and (b) the PALC service. Such research seeks to inform the ongoing development of effective perinatal services.

**Method:**

A qualitative interview-based research design was employed with a purposive sample of 10 mothers who had previously experienced perinatal loss and who attended the Mater Mothers’ PALC during their subsequent pregnancy in 2015. All mothers had subsequently delivered a live baby and were in a relationship with the father of the new baby. Women were aged between 22 and 39 years, primiparous or multiparous, and from a range of cultural backgrounds. Semi-structured interviews, conducted either at the hospital or by telephone by an experienced, independent researcher, lasted between 20 min and one hour. All interviews were audio-recorded and transcribed verbatim, with participant names changed. Interviews were analysed using content analysis by two researchers who were not involved in the service delivery or data gathering process.

**Results:**

Seven themes were identified from the interview material: The overall experience, The unique experience of first pregnancy after loss, Support from PALC, Experiences of other services, Recommendations for PALC services, Need for alternative services, and Advice: Mother to mother.

**Conclusions:**

Participants spoke positively of the PALC services for themselves and their families. Anxieties over their subsequent pregnancy, and the desire for other health professionals to be more understanding were frequently raised. Recommendations were made to extend the PALC service and to develop similar services to support access for other families experiencing perinatal loss.

**Electronic supplementary material:**

The online version of this article (doi:10.1186/s12884-016-1200-9) contains supplementary material, which is available to authorized users.

## Background

It has been estimated that 86% of women become pregnant again within 18 months following perinatal loss [1]. Because a woman’s response to her subsequent pregnancy may be influenced by her grief over her previous perinatal loss [2], specific health care services have been developed to provide support during this potentially difficult period. However, there is little evidence of outcomes from these hospital-based services. The aim of the present study is to understand the experience of mothers who received care from a specialised bereavement and antenatal service during their subsequent pregnancy following perinatal loss.

*Perinatal loss* commonly refers to miscarriage (loss of pregnancy before 20 weeks gestation), stillbirth (death from 20 weeks gestation with a birth weight over 500 g), and neonatal death (loss up to 28 days after birth [3–5]. The focus of this study, however, is on stillbirth and neonatal death. The rates of stillbirth per 1,000 births vary from one in 18.4 births globally, to one in 3.4 births for developed countries [6]. There are 19 neonatal deaths per 1,000 births worldwide [7]. Cumulatively, this highlights the high prevalence of perinatal loss, and while the prevalence is higher in low to middle income countries [8], millions of women are impacted worldwide [9].

These bereaved women commonly experience a range of difficult emotions including distress and hopelessness [10], and have a heightened risk for mental health conditions (e.g., anxiety, posttraumatic stress disorder, and depression) [11, 12]. They may also blame themselves for contributing to the outcome, and perceive that their body has failed them [13, 14]. In addition, support from significant others may be perceived as inadequate or limited, leading to potential isolation [15].

Importantly, the consequences of previous perinatal loss may extend to a subsequent pregnancy [16, 17]. Women who become pregnant again may doubt their ability to maintain a successful pregnancy, and fear a reoccurrence of perinatal loss [13, 18]. They are also at high risk for depression, with Armstrong [19, 20] demonstrating that almost half of the previously bereaved women studied reported depressive symptoms meeting criteria for diagnosis. These women also have increased levels of pregnancy anxiety [21], particularly surrounding birth and significant milestones [22]. Bereaved women may be hypervigilant about the health of the new baby, and emotionally distant to the foetus/baby [23, 24].

To support women immediately after perinatal loss, numerous interventions have been described [15, 25–30]. However, less is known about services during a subsequent pregnancy for women with a history of perinatal loss. Documented services for women during a subsequent pregnancy include: a telephone support service by peer supporters [31], home visits by nurses [32], and perinatal loss support groups [33]. In particular, hospital-based care may be offered in a specialised clinic [34].

### The pregnancy after loss clinic

One such hospital-based service is the Pregnancy After Loss Clinic (PALC), part of the Bereavement Support Service at the Mater Mothers’ Hospital in Brisbane, Australia. This public midwifery-based clinic was developed in response to the unique needs of local women in a pregnancy subsequent to perinatal loss. Women are referred by their general medical practitioner (GP) to the Mater GP liaison midwife early in their subsequent pregnancy (6–8 weeks) and, where previous perinatal loss is identified, the midwife may triage the referral for the PALC. The PALC midwife typically meets with the women between 8–12 weeks gestation. A multidisciplinary team (registrar, sonographer, midwives, counsellor, and consultant obstetrician) then provides emotional and clinical care that is collaborative and attuned to the specific needs of this population during the antenatal and intrapartum period.

All PALC midwives have loss and grief training, and may have met or cared for the parents following the previous perinatal loss, creating a trusting foundation. From the initial visit, a schedule of appointments is determined, based on women’s obstetric and psychological risk factors. Women also have access to antenatal pregnancy after loss parenting classes, a PALC midwife who carries a dedicated mobile phone during normal clinic hours, and 24-h care through the hospital’s Pregnancy Assessment and Observation Unit. Couples can access additional supportive counselling through the bereavement service and individual or couples’ counselling, if required. While a range of individual care plans emerge as a result of these choices, women and other family members typically have 13 scheduled PALC appointments and 2–6 ultrasound appointments during the pregnancy. Although this contact usually ceases prior to delivery, the PALC midwife may visit mothers on the ward after birth, if time permits.

While there are no quantitative studies regarding the effectiveness of such hospital-based interventions in other settings, one qualitative study was located. Caelli et al. [34] used a phenomenological method to explore the experiences of Australian and Canadian women during their subsequent pregnancy following previous perinatal loss, considering the services received from a midwifery-managed hospital-based program. In this program, services included telephone access to midwives, antenatal care, and support during labour. Findings revealed that women experienced intense grief, fear, and anxiety during their subsequent pregnancy, as well as reporting high satisfaction with the support received from the program.

### The present study

The aim of this research was to understand the experience of pregnancy and birth for mothers in a pregnancy following perinatal loss, and to understand their experience of the specialised PALC provided at the Mater Mothers’ Hospital in Brisbane, Australia. It is hoped that this information will support ongoing quality improvement of the PALC, and contribute to the development of other services of this nature.

## Methods

### Methodology

This study employed a descriptive, interview-based, qualitative content analysis design [35, 36] with maximum variation purposive sampling [37].

### Participants

Participants were 10 mothers with a history of perinatal loss (during 2008–2014). While some of the participants’ previous babies were stillborn, about half were alive at birth and lived between two and 24 days in a Neonatal Intensive Care Unit. Most babies were premature, with more than half born before 26 weeks of pregnancy, while two were born at term or later (i.e., 40–43 weeks). These mothers had become pregnant again and been referred to the PALC at the Mater Mothers’ Hospital during 2015, where they subsequently delivered a live baby. All mothers were: in a relationship with the father of the subsequent baby, aged between 22 and 39 years, and from a range of cultural backgrounds including Caucasian, Aboriginal, and Maori. Some mothers had older children while others did not. The focus of this study was on mothers as the admitted patient, and fathers were excluded.

### Data collection

Participants were engaged in a semi-structured interview regarding their experiences, using an interview guide and associated prompts developed for the study (see Additional file 1). Questions were phrased in a way to engender reflection on experience, while permitting participants freedom to control what aspects of their experience are discussed [34]. This is important from an ethical perspective, as research of this nature is highly sensitive and has the potential to trigger distress.

### Procedures

Ethical approval for the study was obtained from both the Mater Health Services (HREC/13/MHS/7) and The University of Queensland (HREC2013000992). Mothers were purposively selected to represent a range of demographic and clinical characteristics, and were invited to participate in the research by one of the PALC midwives. They were provided with information letters and consent forms which were signed and returned prior to interview. Upon return, administrative staff arranged appointments for interview.

Ten semi-structured interviews were conducted by one author (LD), who is an occupational therapist trained in interview techniques and experienced in working with parents. The interviewer was not involved in the PALC and was not previously known to the mothers. Interviews were conducted either in person at the hospital, or by telephone, depending on parental preference. They lasted between 20 min and one hour. All interviews were audio-recorded and transcribed verbatim by a professional transcription service. Names were replaced with pseudonyms.

### Data analysis

Interviews were analysed by two researchers (PM and GB) who were not involved in the PALC or the data gathering process. First, reflexivity was used to enhance trustworthiness of data support and rigour; that is, the researchers who analysed the transcripts made a record of their thoughts and experiences about the topic prior to analysis, to support emergence of potential insights that might influence data analysis [37].

They then independently listened to the audio recordings and read through the transcripts several times, highlighting meaningful text and noting themes emerging from the content. Themes were then tabulated independently by each researcher using the word processing package, Word (PM) or NVivo software (GB), to identify themes/sub-themes/categories. Once each transcript had been analysed, the two researchers met on three occasions to compare and refine themes. A peer check was undertaken by a third researcher (JS) who read these themes and provided additional comments about clarity [38]. Discussions continued until full consensus was attained.

## Results

Seven themes were identified from the interviews, with many reflecting the interview questions: 1) The overall PALC experience, 2) The unique experience of pregnancy after loss, 3) Support from the PALC, 4) Experiences of other services, 5) Recommendations for the PALC, 6) Need for appropriate alternative services, and 7) Advice: Mother to mother (see Table 1 for a summary of the themes and sub-themes, and Additional file 2 Table S1 for a more detailed summary that includes categories). Themes are discussed below.

**Table 1:** Summary of themes derived from thematic analysis of the transcribed interviews

Main theme	Sub theme
1. The overall experience	1.1 Mothers’ perspective
	1.2 For fathers
	1.3 For siblings
	1.4 For other family members
2. The unique experience of pregnancy after loss	2.1 Heightened emotions
	2.2 Triggers
	2.3 Personal growth
	2.4 Emotional restraint
	2.5 Individual journey
	2.6 Ways of coping with bereavement
	2.7 Partner’s experience
	2.8 Siblings’ experience
	2.9 Experience of other family members
3. Support from PALC	3.1 Service characteristics
	3.2 Emotionally supportive relationships
	3.3 PALC care provision
	3.4 Services available
	3.6 Balance between medical and emotional support
4. Experiences of other Services	4.1 Midwives (non-PALC)
	4.2 Doctors
5. Recommendations for PALC	5.1 Extending existing PALC
	5.2 Partner-specific PALC services
	5.3 Child-specific PALC services
6. Need for alternative services	6.1 For mothers
	6.2 For children
	6.3 For partners
7. Advice: Mother to mother	7.1 Address concerns
	7.2 Utilise coping strategies
	7.3 Moving forward

### The overall PALC experience

The mothers were overwhelmingly positive about their experience of the PALC. Adjectives frequently employed included: amazing, reassuring, incredible, brilliant, spectacular, helpful, supportive, excellent, friendly, warm, and “…very very very good” (Nerida). A number of mothers credited the clinic with maintaining their ‘sanity’ during the transition: “I just don’t think I would’ve stayed sane into my pregnancy without it” (Emily).

The mothers similarly noted that their partners benefitted from the service. First, they felt that their relationship with the PALC supported access for the father: “…because I got along so well with [the PALC midwife]…my partner felt quite comfortable calling her as well and asking her questions” (Emily). Second, partners’ needs were catered for when attending appointments: “…they would say to him ‘…And how are you too? Have you been well? How’s work?’, so that he is cared for as well” (Margy). Third, knowing that the mother was receiving specialised care was reassuring for partners: “…[partner] knowing I always had someone to turn to, that made him feel comfortable” (Jane).

Some women perceived that their partners had minimal need of the service: “Well, I personally – I think women understand more than, um, a man would… I’m not saying that, like, my husband wasn’t sad or anything but I just don’t think he really needed that kind of support” (Nerida). Others felt that partners would not want to talk to anyone about their experience. Shyness (Margy) and grief “…[he] doesn’t like to talk about what happened to us too much and I think it’s a bit raw for him still” (Debbie) were given as reasons for this.

Two barriers to partner participation were reported. *Work* was identified as the main barrier: “My husband…he didn’t really, like, have a lot to do with it, because the majority of my appointments would be, you know, during the week and during when he was working” (Wendy). A lack of *knowledge* of services available for partners was identified as a further barrier. Finally, partners’ *selectively attended* appointments they saw as important, such as the orientation, first appointment, and key medical tests: “He’d come to the, you know, the scan, the important ultra-sounds” (Wendy). It was seen as the mothers task to “…relay everything back to him” (Indy) about appointments not attended.

Mothers discussed their caution about including their children in the clinic, but where they did, the PALC midwives were supportive: “…we were thinking about taking them [the children] to the 20 week scan, but because we wanted to make sure that everything was okay, they came to the 24 week one here and…[the PALC midwife] explained everything.” The consultant was also mentioned for his supportive and positive interactions with the children: “[He] got M. (daughter) to do my blood - she took my blood pressure. So, you know, she still talks about having to pump me up…” (Angie)

Finally, mothers appreciated the inclusion of other family members in the service. For example, Karen noted that “It’s not just for parents”, and that her mother had benefited from “…talking to [the PALC midwife] about her concerns”. As in the case of the partners, knowledge that their family member was accessing the PALC was experienced as comforting: “…my family…they sounded extremely reassured to know that I wasn’t going through it alone, that I had support there which really set their minds at ease” (Emily).

### The unique experience of pregnancy after loss

The mothers particularly appreciated the recognition by PALC midwives of the unique characteristics of a subsequent pregnancy following perinatal loss: “Everybody was…understanding that you weren’t just a normal mum anymore, you were a…pregnant mum coming in with a lot of mixed emotions” (Margy). Mothers gave examples of the ways in which the previous loss had exacerbated their worries, anxieties, and fears. Factors that heightened these emotions included medical environments, milestones, and physical sensations. Returning to *medical environments* reminiscent of their previous loss was particularly distressing: “I hated coming to level 9, because that’s… the level where everything happened for me and I just dreaded it” (Margy). The high level of understanding demonstrated by the PALC midwives in this regard was appreciated.

Three participants identified *milestones*, such as the gestational week of the previous loss, as triggers for distress: “I didn’t start getting really nervous until…around 23 weeks, that’s when I started going, oh, is it going to happen again?” (Wendy). Changes in *physical sensations* also contributed to distress: “Just one of those days where I [am] not feeling movements yet…” (Debbie).

Extreme emotions were discussed. Angie was conscious that these *fears* made her look “…like I was this neurotic pregnant woman”, and Brooke recognized that she needed to develop new skills to “…calm me down and keep me relaxed…because I don’t have any of that.” Women also spoke of their *anger* about a number of aspects, including: the loss of the previous baby, their dissatisfaction with other services at that time, the perceived unfairness that other women can have live births, and their lengthy grief journey. *Guilt* over being pregnant again and possibly forgetting the lost baby was also raised.

Perhaps related to these feelings, participants spoke of a *reluctance to embrace their new pregnancy* due to fear of further loss. This was exhibited in terms of reluctance to bond or prepare practically for the baby (e.g., attending medical appointments, asking detailed questions). For example, Wendy stated that both she and her partner had difficulties “attaching” to the baby as they “…didn’t think the pregnancy was going to…go ahead and that we’re going to get babies at the end.” Consequently, they “…didn’t actually set the nursery up, until…a month before they were due”.

Positive insights also emerged. The women evidenced a range of ways in which they *grew and changed* following their experience of loss, including having extra compassion for other women in similar circumstances, and a drive to support other women experiencing loss: “I just want to make it easier for other people too” (Margy).

Despite the commonality of perinatal loss, the mothers spoke about the very *individual nature* of the journey during a subsequent pregnancy:

“…[understanding] each individual person that’s on that journey of a pregnancy after loss, and understanding how they like to be treated and how they like the relationship to go and how they like the information, because… [depending on whether we had a miscarriage or live baby who passed away] we needed to be treated very differently.” (Emily)

The mothers identified a number of strategies to cope with their previous loss and the new pregnancy, with the main topic being *social support*. Most mothers described needing someone to talk to about the loss: “Most of us like to talk about our angel babies.” (Angie). Mothers also reported a general lack of available support and understanding from people closest to them:

“I think even my family, like…even my husband. I don’t think they would understand what I’ve gone through… especially, like, my mother-in-law and my mum, I think they can’t… really understand exactly how I feel with the loss” (Nerida).

Friends were also seen as removed from their grief: “It’s hard when you go out with your other mates and…they’re talking about all the fun stuff. And all you want to talk about is ‘I’m getting a cool headstone for my daughter’” (Margy). “People just kind of want to pretend that it never happened” (Karen).

In relation to the new pregnancy, there were individual differences regarding preferred timing of support, with most mothers wanting support at all stages, while Brooke needed “…really nothing until the end”. Some mothers were reluctant to disclose their new pregnancy: “I didn’t make it common knowledge at work even until after my 20 week scan…” (Angie), potentially contributing to their sense of isolation.

Other coping strategies reported by the women included: gaining *knowledge*, “I can deal with something a lot better if I understand it” (Brooke); taking it *moment by moment*, “You sometimes just have to go breath by breath, minute by minute” (Angie); setting *short-term goals*, “[To] get over that hump and on to the next one… I’d set goals; I got to 12 weeks and then the 20 – so it was like, you know, I set little milestones” (Debbie); *memory boxes*, “…when you leave The Mater [following the earlier loss]… you’re given a memory box, so then you don’t leave the hospital empty handed” (Jane); *making changes*, “…we couldn’t actually go home… so then, we [were] just like, okay, let’s move” (Wendy); and the process of trying to *make meaning* of the loss, “…but I’m like, you know, obviously there’s a reason, we don’t know what it was” (Angie).

The loss of the baby not only impacted the women, but also the partners, other children, and the wider family. While some women perceived that their partners were removed from the loss, others reported that their partners felt “helpless” (Karen), or were still “emotionally raw” (Debbie). During this time, many wished that their partners would obtain support and talk more: “I just wish he’d talk” (Jane). These emotional reactions seemed to carry over into the subsequent pregnancy with partners having difficulty “trusting that things would be alright” (Brooke), and feeling “stressed and hypervigilant” (Karen).

Some mothers noted the impact of the loss on their older children. One child now anticipated a second perinatal loss, rather than a successful birth (Debbie), while another had “a very hard time” (Margy). The child’s social environment may have also affected his/her coping: “M. [son] started prep…and he would talk about L. [lost baby] and people would go, ‘What are you talking about? Your sister is M.’ ‘No, my sister is L.’” (Angie).

Mothers perceived the wider family to be at varying stages of coping with the loss and transition to the new baby. While Nerida indicated that she didn’t think the wider family would “need the extra support”, Karen noted that “…it was our family as well that lost, you know, a niece or a nephew or a grandson”.

### Support from the PALC

Participants spoke openly about the specific aspects of the PALC that they appreciated, including continuity of care, accessibility, availability, flexibility, and regularity. Mothers appreciated developing rapport with consistent care providers, such as sonographers and midwives, who knew their background: “I loved seeing one person all the time so I didn’t have to tell my whole story again” (Debbie). When that health professional was away, participants also felt reassured that others in the team knew their circumstances and could assist if needed: “…anytime [the PALC midwife] wasn’t there we’d see a different midwife… and it was just seamless. It was, kind of, just like meeting another member of the family” (Karen).

The women found the PALC *easy to access* as they were encouraged to call, text, or email (according to their preference) midwives directly with any questions or when in need of support. Even just the *availability* of the service was a reassuring presence: “I didn’t [call or email], but the thought, you know, that I could do it was reassuring” (Debbie).

The mothers also appreciated the *flexibility* of the service. In addition to routinely-scheduled appointments, there was capacity for lengthier and additional appointments: “We felt really comfortable in staying and talking even if it meant our appointment went over [time]” (Karen), and “…[the midwife] would listen and if, um, I thought something was wrong with bubs… she would just…pretty much, just say, ‘Oh, well, just come in get bubs checked out’, you know, just to put my mind at ease.” (Wendy). However, Indy observed that “…the clinic…was only, sort of, on one day [of the week], so, I guess, there wasn’t much flexibility with that”.

Mothers appreciated the *regularity* and frequency with which the PALC maintained contact: “I did get regular phone calls…just saying, ‘Oh, how are you doing?’…‘How’s bub? Are you feeling well, and how are you emotionally?’” (Nerida). Angie noted that, with her range of appointments (PALC midwives, cardiotocography/CTG, consultant), she was attending the clinic almost weekly when needed.

In addition to the service characteristics that were valued by the mothers, they spoke frequently about the *emotionally supportive relationships* developed with the PALC midwives. During their interactions, the mothers valued the: rapport developed, active listening skills, caring nature, and realistic attitude. For instance, Emily reported that the staff gave her the:

“…feeling that you’re there, kind of, talking to a friend, someone who really does genuinely care about you and about your pregnancy and about the baby that you’ve lost…not being that overly optimistic, bubbly, not afraid to talk about the realistic situation.”

Parents also spoke of the *validation* they experienced: “What [the PALC midwife] did very well was she’d let you know that the feelings you were experiencing were experienced by other parents going through the same journey.” (Emily). As noted by Karen: “The most helpful [thing]…was just knowing that what had happened with us in the past was taken seriously, and that it was kind of incorporated into this pregnancy.” Additionally, the feeling of being *understood* was appreciated: “The support from PALC clinic is very, very helpful because I think they do understand what I’m going through, not like other people” (Nerida).

Mothers noted the PALC midwives’ capacity to *anticipate* what they might experience: “Um, she’d also let you know…what emotions you might start experiencing and what things to, kind of, be aware of” (Emily). This relationship also instilled *hope* that their new pregnancy might be successful, and re-energised them: “…I actually went out of it [meeting with PALC midwife] feeling good, not feeling drained or exhausted as I thought I was going to be” (Brooke).

In terms of the *provision of care* from PALC, the women were cognisant that the staff made every effort to *tailor* the care to their needs and preferences: “She very much worked around what I felt I needed and how I wanted the pregnancy and birth to go and was as accommodating as she could be towards that” (Indy). Where possible, women perceived that they were matched with other staff based on factors such as previous contact, demeanor, and personal preferences.

The women perceived that the PALC team regularly *advocated* on their behalf as the midwives would “… listen to what I need and help that I get it” (Nerida). Alternatively, Angie noted that the PALC midwives empowered the women to advocate for themselves: “…[the midwife] was like, ‘Well, no. You need to stand up for what you need’” (Angie). In this process, the *streamlining of services* was observed on most occasions, with improved access to the additional support and medical assessments needed:

“Yeah, like if I felt something was wrong, [the PALC midwife] would pass [this] onto the nurse and to the doctors and then I’d come in to see the doctor, like, they would have that message and then they’d, like, check up on it.” (Wendy)

The women spoke of the nature of the *services available* to them through the PALC, including education, specialised equipment, and access to extra services. They felt that any questions they had were clearly answered in language they could understand. Women spoke of the comfort they derived from the scans, and particularly of their extra access to these, which was perceived as being beyond that of a typical pregnancy:

“…it was really interesting ringing downstairs to the assessment unit and you would say, ‘I want to come in for an extra CTG’. [The assessment unit would reply] well, basically, ‘Why?’ … ‘Well, I’m part of the Pregnancy After Loss Clinic.’ ‘Okay, come in whenever you want.’” (Angie)

Importantly, the women indicated that, while both *medical and emotional care* were critical to their pregnancy, it was the emotional care provided by the PALC that was most valued. Emily: “I felt like my clinical and my emotional care were both very good…And my memory of it is more the emotional support being the thing that really stood out to me.” Participants spoke mostly of having their clinical needs met by the doctors and midwives, and their emotional needs met by PALC midwives.

### Experiences of other services

Participants spoke of the broader experience of pregnancy and birth, particularly their contact with midwives and doctors in different services at the Hospital, including the birthing suite and the assessment unit. For example, Angie noted:

“I know some of the midwives downstairs in the assessment unit can be a bit funny sometimes. Not blasé but just sort of, ‘Oh no, everything is fine, that should be happening…’ …and I know that they’re trying to reassure me as well, so it’s just their manner and personality.” (Angie)

The mother’s experience with the *doctors* was discussed at length, with several subthemes emerging. Mothers often commented on the importance of their *relationship* with their doctors, and especially valued having doctors they knew from previous experiences and who *understood their previous loss and the associated anxiety*:

“Yeah, [the doctors] were really good and understanding because…I was, you know, quite freaked out a lot and they understood how freaked out I was. They just, you know, listened and… it seemed like they were caring about the whole pregnancy.” (Wendy)

When meeting new doctors, mothers reported that they: “…have to try and get that trust with them after what we went through…it took me, you know, a while to get their trust, but yeah, they eventually got it” (Wendy). To this end, the need to see the same doctor over time was highlighted: “…having the same [consultant], even though I saw him probably maybe four or five times, it was still nice to build that rapport with him so that when I did go into spontaneous labour, his team was here” (Angie).

In contrast, not knowing the doctors led to concerns that they may not adequately manage the pregnancy and address medical needs. For example, Emily found that a doctor taking the place of her usual doctor “…was quite young and he was quite indifferent to what I had been through, and …didn’t have knowledge of my medical condition that, you know, could possibly lead to complications.” Karen noted: “…the first doctor that we saw…um, he didn’t open our file before we walked into the room.”

Participants valued the ability of many of the doctors to answer questions in plain English: “It was really nice to speak to a doctor that used English instead of doctor-talk” (Karen). Mothers also noted that it was not always easy to ask their questions, with some doctors perceived to be intimidating:

“…you think of all these things that you want to ask all the doctors on ward round, and as soon as they walk in you, sort of… back down, because they’re, like, they’re higher up…So you, sort of sit back a bit in your chair and lose your voice and…they come in and chuck all their medical jargon at you and their thoughts and theories and you, sort of, you process all that and you forget what you need to ask and yet, I did write down things.” (Jane)

Participants expressed frustration when previous conversations and care plans were not known to others on the team:

“You know, I’d discussed with [the consultant] very early in the pregnancy about if the pregnancy was breach and he didn’t turn, that we wouldn’t even attempt to turn him manually… and yet on my last appointment with this [different] doctor he was asking me if I wanted to try and have the baby turned. And I’m like, ‘Well, no, we’ve discussed this,’ and then he had to call and ask if was okay that they didn’t try.” (Emily)

Two mothers reported specific concerns about their interactions with doctors – one in which the doctor had accessed the incorrect file, and another in which the doctor was perceived to inappropriately discuss aspects of the service, such as the funding model.

### Recommendations for the PALC

Included in this theme are ideas for extending the existing services, with responses grouped into several sub-themes: pre-conception, antenatal, intrapartum, and post-natal care. Indy noted that previous perinatal loss is compounded when women have difficulty conceiving, and that recognition of this interface *pre-conception* would be valuable.

*Antenatally*, participants generally wanted more contact with PALC. They suggested more access to midwifery appointments and tests: “I think it’s just more peace of mind really” (Karen), and to extend PALC availability to additional days. They requested that PALC midwives visit antenatal inpatients more regularly, and attend other antenatal hospital appointments to provide emotional support and streamline communications, to: “…kind of, bridge that gap if you do have a change of doctor” (Emily).

Karen recommended ensuring that family members can access support from the midwives: “Maybe just providing a number that they could call and, you know, somewhere where they can voice their concerns.” It was suggested that more midwives should be employed: “More staff would be awesome because …sometimes it’s harder to get in for check-ups and stuff” (Angie).

Another recommendation was to introduce expectant parents to the delivery midwives:

“…when you came to the actual birth, it was, sort of, pot luck what midwives were on… it would have been nice to have perhaps known who was going to be there at the birth… thinking, ‘Oh, I’m not going to know anyone here, are they going to know my background?’” (Indy)

During the *interpartum* period, mothers expressed a preference for having the PALC midwife, with whom they had developed rapport, in the birth suite with them: “…it would have been nice to have the midwife that we had throughout the whole pregnancy, be there for the birth as well” (Karen). Some had even assumed this to be the case (e.g., Wendy).

Despite having access to other midwives *postnatally*, mothers expressed a desire to continue contact with the PALC midwives: “I would have loved to have been able to come at six weeks and go, ‘Look at my chubba bubba’” (Angie). Indeed, some mothers indicated that they *had* made these contacts: “…because it’s, sort of, so nice to come in and see [the PALC midwife] and I’ve emailed her a few times afterwards” (Indy), and “…even after the pregnancy…I’ve rung [the PALC midwife] just to question about babies and she’s been quite happy to help” (Karen).

Indy indicated that this would be valued as bereavement issues continued to arise after delivery. She recommended:

“…checking in with people just to see if, you know, they’re having problems, suffering post-natal depression or anything…I think a lot of emotion would come up because, you know, certainly since having [the baby] has made me realise how much we missed out by not having B.…so, yeah, so it definitely brings up quite a lot of emotion.”

Finally, Angie observed that “…personally, I built up such a rapport that I miss them [midwives]”.

Mothers also wanted to *develop a better relationship with the doctors* and to obtain *more individualised care.* Jane suggested “…a little bit of a get to know you”, to “…voice to them your thoughts and… your anxious moments and what worries you… [so] they sort of just remember you a bit more, as opposed to having to always check back on your clinical notes.”

The mothers made recommendations for a range of additional PALC *support* options to complement existing supports, including PALC-specific support groups for mothers and fathers interested in meeting face-to-face at all stages of the pregnancy and afterwards, a PALC online support group or forum, and a PALC Facebook page. Jane recommended a dedicated PALC “24 h hotline” (as opposed to the general hospital hotline) to alleviate anxiety “…because you would have random 2.00 am wake ups and go, ‘Oh, my God, bub is not moving as much’.”

Women also wanted the PALC team to *provide recommendations* for appropriate support groups, and particularly for ones in their area:

“I know that there are ones [support groups] available but sometimes you don’t want to sit down and have to search the Internet and try and find them. So maybe if there was a support group that they were affiliated with…” (Emily)

Emily recommended that PALC establish a “*sponsor”* or a *“mentor”* program, such as in Alcoholics Anonymous: “…there’s nothing you can say to make someone feel better… but it’s nice to be able to talk to somebody who’s been in the same shoes as you” (Karen). Such a program might have advantages for both the mentor and mentee:

“I liked…feeling like you could help somebody that was going through something so terrible… you’re always feeling like you have to be so strong, and to have someone that you can just be very honest with and break down if you want to break down.” (Emily)

In addition, women suggested providing access to *information support* in the form of books, orientation information, classes, and “…more online information” (Brooke):

“Facebook and Google aren’t exactly a wealth of correct information so maybe some articles…about what you’re going to come up against and…more towards the whole birth side of it…ways of dealing with stress… tips for wellbeing and positive thinking… calming techniques.” (Brooke)

Mothers commented on having received and valued a book from the PALC called “Someone Came Before You” [39]. Access to additional *books*, and particularly to books offering a range of cultural and religious perspectives, was recommended. Others suggested a need for additional *orientation information* relating to the roles of professionals in PALC, and what to expect.

While some women felt that their partners and children did not require a service from the PALC, others recommended developing *partner- and child-specific PALC services*: “…he came to a few of my appointments; I suppose he was saying he didn’t feel like there was a lot for males” (Indy). Angie recommended that PALC might also make it clear whether or not it is appropriate to call them about issues with older children, and perhaps “…pre-empt [questions from children] and go, ‘Well, you know, you’re likely to get these questions.’ [Children] come out with stuff.” Mothers especially wanted guidance from the PALC on alternative services that had a good understanding of loss and bereavement and were appropriate to access for their partners and children of various ages.

### Need for appropriate alternative services

While mothers felt that the PALC met their needs, they were aware that such a service was not available in other geographical areas. A number of the women recommended that new PALCs be developed in other locations, perhaps even making it “…a standard thing” (Indy) in all hospitals. This was particularly noted by mothers who came from outside the usual catchment area of the Mater hospital.

Mothers sought alternative means of support from outside the PALC, including online support groups (e.g., Rainbow Connection, Supporting Mothers with Angel Babies), perinatal associations (e.g., Still Birth and Neonatal Death Association), and mentoring. Brooke valued “…knowing that other people were going through the same thing, that it wasn’t, sort of, me, singled out…I’m glad I’m not the only one.” While mothers appreciated the advice from other mothers through online support groups, they were frustrated in their search for appropriate support near to them: “I tried to go on Facebook but a lot of the groups were American, so there wasn’t [any], not that I found anyway, in my area” (Debbie).

For some mothers, support groups were seen as useful only for a specific period of time:

“There’s a group [online] called Mums Like Me and it’s for mums who’ve had a loss of some sort, whether it’s stillbirth or whatever, and…I haven’t been able to be back on since I found out I was pregnant because…some of them are still so bitter and twisted about stuff …[I] can’t afford to get into that stuff all the time.” (Angie)

Where mothers observed that the PALC service was not designed to meet the needs of their partners, children, and other family members, they highlighted a need for access to alternative services, and were unsure if these services existed.

### Advice: mother to mother

Mothers’ key recommendations to other women who might be going through a similar experience were to: address their concerns, use a range of coping strategies, and continue to move forward. In particular, they advised mothers to advocate for themselves and not to feel intimidated, especially with doctors, and to: write/ask questions, make additional appointments, get second opinions, have additional tests, keep looking until you find professionals you trust, and stay in control of the pregnancy and related decisions. Women were also advised to trust their instincts and act accordingly, with examples given of how these instincts had so often proven to be correct.

In terms of specific *coping strategies*, the value of *social support* and the need to ask for help, talk about feelings, go online, meet other mothers, and take or leave advice as needed, was emphasised. The benefits of *journaling*, or blogging, in the form of writing events, thoughts, feelings, questions, and even letters to the baby, were mentioned.

Mothers recognised that the idea of *moving forward* could be challenging and wanted others to know that “…it gets easier” (Wendy), “…nothing lasts forever… and you’re not alone” (Margy). They recommended that mothers “take each day at a time” (Indy), with a “breath by breath, minute by minute” approach (Angie). In particular, mothers wanted others to know that *it is okay to move on,* “…sometimes you think that you might start to forget [the lost baby] them but then you’re like, it’s not you forgetting them, like, you’re moving on.” (Wendy); “We all have to move on, but be happy again” (Margy). Karen advised mothers to “embrace” the new pregnancy: “…try to be excited, try not to let past experiences taint this new experience.” Other mothers recommended: setting small achievable goals or milestones, to have “hope”: that “…it’s a different pregnancy, different baby…” (Debbie), and to return to previous occupations such as “…getting back to the job [at a school] and seeing all the kids again.” (Wendy)

## Discussion

This study is the second to investigate women’s experience of a specialised hospital-based clinic for pregnant women following previous perinatal loss. Consistent with earlier work [34] experiences of this service were overwhelmingly positive. Mothers highly valued the PALC service for themselves, and perceived that their partners, children, and other family members also benefited, both directly and indirectly.

Mothers provided information both reinforcing and advancing existing literature. The unique nature of pregnancy following loss was highlighted, including mothers’ heightened emotions [10–12, 19–21, 34] and hypervigilance [23, 24]. Consistent with the work of Côté‐Arsenault and Donato [22], milestones, such as the gestational age of the previous loss, and a perception of altered/decreased foetal movement, were particular triggers for distress. Also consistent with previous literature [40, 41], mothers preferred consistent caregivers who were familiar with their prior experiences, plans, and birthing decisions. This diminished their distress and improved feelings of security [41].

In accordance with earlier research [15], mothers in the present study felt that support from significant others outside of the PALC was limited, leading to potential isolation. They reported accessing a range of coping strategies, drawing on both the PALC and other specialised avenues, and desired further guidance to learn about appropriate supports. A number of improvements were recommended, both within the PALC and for related services, to facilitate access to these supports. Recommendations included improved knowledge and clarity about available services, increased service flexibility, and greater and more varied informational resources. In response to a similar need, Smart and Smith [42] developed an information booklet called “A Love Not Forgotten”. Access to books offering a range of cultural and religious perspectives was recommended.

Importantly, some mothers expressed hesitancy bonding with the new baby. O’Leary [43] has previously noted that grief from earlier perinatal loss can re-surface during subsequent pregnancies, and observed that mothers may either be more detached or more diligent and overprotective toward the subsequent unborn child. As poorer ante-natal attachment has been associated with more maladaptive outcomes (see [44]), interventions to support the parent-foetal relationship in the subsequent pregnancy may be warranted (see [17, 43]).

Mothers in the present study disclosed concerns not only for their new pregnancy, but also were mindful of the needs of their partner, children, and other family members. Relatively little research (see [40]) has addressed the impact of perinatal loss on siblings [45, 46] and other family members [47, 48] during a subsequent pregnancy. This care-giving burden can represent an emotional load for bereaved mothers, and warrants consideration in treatment programs for these families.

The unmistakable value mothers endowed their relationship with PALC midwives, together with their efforts to continue this relationship following birth, suggests a need to formally terminate the therapeutic nature of this relationship. As a time-limited service, the PALC should consider planning for termination from the initial stages of orientation.

Finally, a number of the mothers interviewed indicated a desire to support other mothers in a subsequent pregnancy following perinatal loss, and were able to suggest strategies to support their journey. This suggests a form of post-traumatic growth by which, over time, people learn to cope with and grow from their experience of grief (see [49]).

### Considerations

While largely reflective of the broad literature in this field, results of this study depict the experiences only of those women interviewed and pertain only to this service. It should also be noted that, while some information was revealed about the partner and other family members, this was from the perspective of the mother, and the partner’s perspective was not actively sought. With knowledge that men and women show different patterns of grief following perinatal loss [4], further studies should directly consider the needs and experiences of fathers. Future studies might also consider larger representative samples and consider quantitative outcomes such as maternal or paternal mood, and relationship with the baby.

## Conclusions

The interviews revealed consistent themes and some individual differences between mothers in their subsequent pregnancy following perinatal loss. The overall experience of these mothers’ of PALC was extremely positive, and participation in the Clinic returned a range of favourable outcomes. Support for anxieties over their subsequent pregnancy, and the desire for other health professionals to be more understanding, were frequently raised. This research sought to inform the ongoing development of effective perinatal services, and the interviews highlighted areas for growth and expansion of the PALC, and of other services, for families experiencing a pregnancy after loss. This study highlights a need for more evidence of outcomes from specialised pregnancy after loss services, including the experience of fathers.

## Additional files

Additional file 1: Interview guide and associated prompts developed for the study. This file contains a summary of the questions used by the interviewer to guide qualitative interviews with participants. (DOCX 13 kb)
Additional file 2: Table S1. Detailed summary of themes derived from thematic analysis of the transcribed interviews. This file contains the more extended details of themes, sub-themes and categories. (DOCX 22 kb)

## Abbreviations

CTG: Cardiotocography
PALC: Pregnancy After Loss Clinic
