# Supplementary material for: “Not just a normal mum”: a qualitative investigation of a support service for women who are pregnant subsequent to perinatal loss
Source: BMC Pregnancy Childbirth. 2017 Jan 5;17:6. doi: 10.1186/s12884-016-1200-9 (PMC5217635; doi:10.1186/s12884-016-1200-9)
Supplement: Additional file 1: — Interview guide and associated prompts developed for the study. This file contains a summary of the questions used by the interviewer to guide qualitative interviews with participants. (DOCX 13 kb) [file 12884_2016_1200_MOESM1_ESM.docx]

**Supplementary file 1**

Interview guide and associated prompts developed for the study:

• What was your general impression of the care you were provided in PALC? What about the care from the midwife? What about the care from the doctor(s)?

• Can you please tell me about the specific aspects of the care in PALC that was helpful? What was helpful and how?

• Was the balance between good clinical care and emotional support achieved?

• What specific aspects of the care provided to you and your baby in PALC could have been better/improved?

• What was the most helpful aspect of attending PALC?

• How well did PALC support your partner?

• How could we further assist women in a subsequent pregnancy in the future?

• What advice would you give other parents going through a subsequent pregnancy?
