# Supplementary material for: “Not just a normal mum”: a qualitative investigation of a support service for women who are pregnant subsequent to perinatal loss
Source: BMC Pregnancy Childbirth. 2017 Jan 5;17:6. doi: 10.1186/s12884-016-1200-9 (PMC5217635; doi:10.1186/s12884-016-1200-9)
Supplement: Additional file 2: Table S1. — Detailed summary of themes derived from thematic analysis of the transcribed interviews. This file contains the more extended details of themes, sub-themes and categories. (DOCX 22 kb) [file 12884_2016_1200_MOESM2_ESM.docx]

**Supplementary file 2**

Supplementary Table 1. Summary of themes derived from thematic analysis of the transcribed interviews.

| **Main theme** | **Sub-theme** | **Categories** |
| --- | --- | --- |
| 1. **The overall experience** | 1.1 Mothers’ perspective |  |
|  | 1.2 For fathers | 1.2.1 Benefited from PALC support of partner |
|  |  | 1.2.2 Different levels of need s |
|  |  | 1.2.3 Access issue |
|  | 1.3 For siblings |  |
|  | - 1. For other family members | 1.4.1 Involvement in PALC |
|  |  | 1.4.2 Comfort from mother’s involvement with PALC |
| 1. The **unique experience** of pregnancy after loss | 2.1 Heightened emotions: | 2.1.1 Worry |
|  |  | 2.1.2 Anxiety |
|  |  | 2.1.3 Anger |
|  |  | 2.1.4 Grief |
|  |  | 2.1.5 Guilt |
|  | 2.2 Triggers | 2.2.1 Environment |
|  |  | 2.2.2 Milestones |
|  |  | 2.2.3 Physical sensations |
|  | 2.3 Personal growth (akin to post traumatic growth) | 2.3.1 Compassion/ empathy / altruism |
|  |  | 2.3.2 Changed from the experience |
|  | 2.4 Emotional restraint |  |
|  | 2.5 Individual journey |  |
|  | 2.6 Ways of coping with bereavement | 2.6.1 Social support |
|  |  | 2.6.2 Knowledge |
|  |  | 2.6.3 Moment to moment |
|  |  | 2.6.4 Short-term goals |
|  |  | 2.6.5 Memory boxes |
|  |  | 2.6.6 Making changes |
|  |  | 2.6.7 Meaning making |
|  | 2.7 Partner’s experience | 2.7.1 Talking |
|  |  | 2.7.2 Feelings |
|  |  | 2.7.3 Unique experience to mother |
|  | 2.8 Siblings’ experience | 2.8.1 The sibling experience of loss |
|  |  | 2.8.3 Emotional sequelae |
|  |  | 2.8.4 Other people’s understanding |
|  | 2.9 Experience of other family members |  |
| 1. **Support** from PALC | 3.1 Service characteristics | 3.1.1 Continuity of care |
|  |  | 3.1.2 Accessibility |
|  |  | 3.1.3 Availability |
|  |  | 3.1.4 Flexibility / timing |
|  |  | 3.1.5 Regularity |
|  | 3.2 Emotionally supportive relationships | 3.2.1 Rapport |
|  |  | 3.2.2 Authenticity |
|  |  | 3.2.3 Acceptance |
|  |  | 3.2.4 Validation |
|  |  | 3.2.5 Caring |
|  |  | 3.2.6 Understanding |
|  |  | 3.2.7 Anticipating needs and emotions |
|  |  | 3.2.8 Hope |
|  |  | 3.2.9 Active Listening |
|  |  | 3.2.10 Responsivity |
|  |  | 3.2.11 Reenergising |
|  |  | 3.2.12 Realistic |
|  | 3.3 PALC Care provision | 3.3.1 Tailoring care |
|  |  | 3.3.2 Advocacy |
|  |  | 3.3.3 Empowerment |
|  |  | 3.3.4 Streamlined care journey |
|  | 3.5 Services available | 3.5.1 Education |
|  |  | 3.5.2 Equipment |
|  |  | 3.5.3 Access to extra services |
|  | 3.6 Balance between medical and emotional support | 3.6.1 Professional roles |
|  |  | 3.6.2 Relative value |
|  |  | 3.6.3 Importance of both |
| 4. Experiences of other Services | 4.1 Midwives (non-PALC) |  |
|  | 4.2 Doctors | 4.2.1 Doctor-patient relationship |
|  |  | 4.2.2 Doctor-patient interactions  4.2.3 Service provision |
|  |  | 4.2.4 Sensitivity to previous loss |
|  |  | 4.2.5 Professional conduct |
| 5. **Recommendations** for PALC | 5.1 Extending existing PALC | 5.1.1 Pre-conception  5.1.2 Antenatal |
|  |  | 5.1.3 Intrapartum |
|  |  | 5.1.4 Postnatal |
|  |  | 5.1.5 Additional services |
|  | 5.2 Partner-specific PALC services |  |
|  | 5.3 Child-specific PALC services |  |
| 6. Need for appropriate **alternative services** | 6.1 For mothers | 6.1.1 Expanding PALC services |
|  |  | 6.1.2 Additional supports |
|  | 6.2 For children |  |
|  | 6.3 For partners |  |
| 7. **Advice:** Mother to mother | 7.1 Address concerns | 7.1.1 Actively advocate for yourself |
|  |  | 7.1.2 Ask questions |
|  | 7.2 Utilise coping strategies | 7.2.1 Support |
|  |  | 7.2.2 Journaling |
|  | 7.3 Moving forward | 7.3.1 “It gets easier” |
|  |  | 7.3.2 It's okay to move on |
|  |  | 7.3.3 Embrace the pregnancy |
|  |  | 7.3.4 Set goals |
|  |  | 7.3.5 Have hope |
|  |  | 7.3.6 Return to previous occupations |
